# Effect of Engineered Nickel Oxide Nanoparticle on Reactive Oxygen Species–Nitric Oxide Interplay in the Roots of *Allium cepa* L.

**DOI:** 10.3389/fpls.2021.586509

**Published:** 2021-02-09

**Authors:** Indrani Manna, Saikat Sahoo, Maumita Bandyopadhyay

**Affiliations:** ^1^Department of Botany, CAS, University of Calcutta, Kolkata, India; ^2^Department of Botany, Krishna Chandra College, Hetampur, India

**Keywords:** nitric oxide, reactive oxygen species, engineered nickel oxide nanoparticle, stress markers, cytotoxicity

## Abstract

Scientists anxiously follow instances of heavy metals augmenting in the environment and undergoing bioaccumulation and trace their biomagnification across food webs, wary of their potent toxicity on biological entities. Engineered nanoparticles supplement natural pools of respective heavy metals and can mimic their effects, exerting toxicity at higher concentrations. Thus, a thorough understanding of the underlying mechanism of this precarious interaction is mandatory. Most urban and industrial environments contain considerable quantities of nickel oxide nanoparticles. These in excess can cause considerable damage to plant metabolism through a significant increase in cellular reactive oxygen species and perturbation of its cross-talk with the reactive nitrogen species. In the present work, the authors have demonstrated how the intrusion of nickel oxide nanoparticles (NiO-NP) affected the exposed roots of *Allium cepa*: starting with disruption of cell membranes, before being interiorized within cell organelles, effectively disrupting cellular homeostasis and survival. A major shift in the reactive oxygen species (ROS) and nitric oxide (NO) equanimity was also observed, unleashing major altercations in several crucial biochemical profiles. Altered antioxidant contents and upregulation of stress-responsive genes, namely, *Catalase*, *Ascorbate peroxidase*, *Superoxide dismutase*, and *Rubisco activase*, showing on average 50–250% rise across NiO-NP concentrations tested, also entailed increased cellular hydrogen peroxide contents, with tandem rise in cellular NO. Increased NO content was evinced from altered concentrations of nitric oxide synthase and nitrate reductase, along with NADPH oxidase, when compared with the negative control. Though initially showing a dose-dependent concomitant rise, a significant decrease of NO was observed at higher concentrations of NiO-NP, while cellular ROS continued to increase. Modified K/Na ratios, with increased proline concentrations and GABA contents, all hallmarks of cellular stress, correlated with ROS–NO perturbations. Detailed studies showed that NiO-NP concentration had a significant role in inducing toxicity, perturbing the fine balance of ROS–NO, which turned lethal for the cell at higher dosages of the ENP precipitating in the accumulation of stress markers and an inevitable shutdown of cellular mechanisms.

## Highlights

- NiO-NP entry into plant cells exhibits dose dependency, and at higher concentrations, NiO-NP display bulk Ni-like characteristics inducing widespread membrane damage before entry into the cytoplasm of exposed cells.
- There is remarkable upregulation of stress-related (APX, CAT, SOD, and RCA) genes in the aftermath of NiO-NP entry into the cells.
- There are significant alterations in cellular hydrogen peroxide and nitric oxide contents, leading to crucial changes in physiological and biochemical parameters of the affected cells due to perturbed ROS–NO homeostasis.

## Introduction

Heavy metals, viz., lead, mercury, arsenic, nickel, chromium, cadmium, and copper, among others, are members of the periodic table with atomic masses of more than 20 and are called as such as their densities are at least five times more than that of water (Tchounwou et al., 2012). Among them, *redox-active metals*, like chromium and copper, can directly affect the reactive oxygen species (ROS) framework of a cell by spontaneously creating an oxidative burst, while *non-redox metals*, like cadmium, nickel, and mercury, induce toxicity either by blocking the functional groups or by replacing cations from essential biomolecules (Schützendübel and Polle, 2002). Though found only in trace amounts on the earth’s upper layer (ranging from minute ppb to 10 ppm), anthropogenic activities, compounded by overpopulation, indiscriminate use of fossil fuels, and spillage of domestic as well as industrial wastes into the environment have led to a disproportionate accumulation of these heavy metals in soil, air, and water, aggravating their percolation into the earth’s crust. Interestingly, plants, the primary producers of our ecosystem, are resilient to increased concentrations of heavy metals as they have evolved a unique strategy of hyperaccumulating and containing them within vacuoles or attaching them to cell walls and concentrating most of the heavy metal ions into the apoplast of the cell (Vázquez et al., 1992).

In the last few decades, many of these heavy metals have found novel uses as nanoparticles in a range of industrial applications and to increase crop yield and production (Rizwan et al., 2019), bringing in a newer and probably more sinister aspect in the heavy metal pollution spectrum (Mustafa and Komatsu, 2016), leading to the constant exposure of flora to these intruding nanoforms widely distributed in soil, water, and aerosol. Like their bulk forms, heavy metal nanoforms too can enter plants quite easily using endocytosis, *via* pore formation and the plasmodesmata, or by hitchhiking on carrier proteins (Pérez-de-Luque, 2017), helped by absorption in lower pH (Järup, 2003). These augment uptake and cellular sequestration of metallic nanoparticles, which then intrude into food webs *via* the primary consumers, and the prospect for their biomagnification through the food chain constitutes a serious hazard to human health. The role of plants in the uptake and biomagnification of potent toxins in the food chain is a well-accepted fact and, thus, needs detailed investigation (Choudhury, 2019).

Nickel pollution has grave consequences on human health, triggering respiratory troubles and cardiovascular issues, as well as certain types of cancers (Genchi et al., 2020). This has prompted imposition of stringent measures against occupational hazards due to nickel dust exposure^1^. Nickel and nickel compounds are graded in group 2B and 1 among carcinogens^2^. However, nickel oxide nanoparticles (NiO-NP) are increasingly being used in heavy industries and consumer products, especially in smelting, iron and steel, coinage, and electrical goods industry (Danjumma et al., 2019), but escape sanctions of scientific and environmental watchdogs. Unplanned disposal of NiO-NP through industrial or consumer wastes precipitates in their spillage into soil and water systems. An essential trace metal for plants, nickel is taken up easily. Even so, a surfeit of nickel inside plant cells can create an upsurge of ROS, which ultimately compromises their survival *via* extensive physicochemical and genomic damages (Manna and Bandyopadhyay, 2017a; Rastogi et al., 2017; Pinto et al., 2019). Plant systems are, thus, particularly vulnerable to sudden fluxes of NiO-NP in soil, water, or aerosol (Ovais et al., 2020). *Allium cepa* (*A. cepa*), the acclaimed ecotoxicological model plant, not only provides the opportunity to study the toxicological effects of environmental pollutants but also can be used to assess the risk of magnification, as it is cultivated widely to be consumed even in raw and unprocessed forms (Boros and Ostafe, 2020).

The by-products of plant metabolism—nitric oxide (NO), a diatomic free radical gas, and ROS, like superoxide anion (O_2_^∗^) or hydrogen peroxide (H_2_O_2_)—are integral parts of the central signaling network in plants and act synergistically in the regulation of crucial processes (Černý et al., 2018). ROS are consistently produced in chloroplasts, mitochondria, and peroxisomes and, in excess, are reactive toward DNA, protein, and lipids. NO reacts with superoxide anions to form peroxynitrites that affect posttranslational protein modifications like S-nitrosylation (Lin et al., 2012). NO also regulates superoxide and H_2_O_2_ production, as well as the function of antioxidant enzymes *via* S-nitrosylation or nitration, thus maintaining an intensive cross-talk with ROS (Romero-Puertas and Sandalio, 2016). Their roles in response to environmental stresses have been investigated for quite some time now (Scheler et al., 2013). Interestingly, there are contrasting reports on the ROS–NO interactions in stressed plants, with both beneficial and disadvantageous outcomes (Farnese et al., 2016). NO can help alleviate heavy metal stress, as reported in cases of cadmium, aluminum, arsenic, and copper exposures (Nabi et al., 2019). Similar observations were reported in instances of exposures to silver and zinc oxide nanoparticles (Tripathi et al., 2017a,b). High concentrations of intracellular NO or changes in their distribution on the other hand initiated unfavorable outcomes in the plant (Domingos et al., 2015). Excess of NO compromised photosynthetic efficiency and inhibited electron transport chains, through the formation of chloroplast-damaging peroxynitrites, impairing many downstream processes in the chloroplast (Procházková et al., 2013).

Pathways of NO production *in planta* are complicated and actively debated. Among the two routes of NO production identified so far are the reductive pathway (primarily executed by the peroxisomal and membrane-bound reductases) and the oxidative pathway (including those involving the hydroxylamines and polyamines) (Farnese et al., 2016). Cytosolic nitrate reductase (NR), otherwise involved in nitrogen assimilation, pitches in for the reductive cascades of NO production, especially during developmental programs or stress management. At the same time, the oxidative route of NO formation is mediated by a nitric oxide synthase (NOS)-like protein complex (Astier et al., 2018). Many components of this vital relationship controlling endogenous NO production, especially during heavy metal exposure, remain to be elucidated.

In this manuscript, the authors have hypothesized that ROS and NO play a causal relationship as stress builds up in freshly rooted *A. cepa* bulbs upon treatment with NiO-NP. A thorough study into the internalization mechanism and the general biochemical and physiological parameters confirmed increased generation of ROS and NO in tandem with increasing engineered nanoparticle (ENP) exposure. NO, initially at low levels, played a benign role. However, on a further increase in NiO-NP dosage, it surged with tandem ROS production. At higher concentrations, NO accumulation became detrimental to cell survival, contributing to a total shutdown of cellular activities. Cellular stress markers like proline increased, inducing prominent changes in potassium:sodium (K/Na) ratio and loss in cell viability, as well as an increase in membrane lipid peroxidation, along with a simultaneous increase in antioxidant enzyme activities, all of which portray extensive physiological and biochemical perturbations of the affected tissue under NiO-NP-stressed conditions. The authors have studied both the NR-centric and NOS-like NO pathways in tissues subjected to increasing doses of NiO-NP. The complex interrelationship between ROS and NO and also their interaction with increasing NiO-NP doses were assessed over a range of ENP doses. The addition of compounds like sodium pyruvate, a H_2_O_2_ blocker, and cPTIO [2-(4-carboxyphenyl)-4,4,5,5-tetramethylimidazoline-1-oxyl-3-oxide potassium salt], a NO blocker, used in combinations or singly, helped to study the dynamics of *in situ* NO on H_2_O_2_ formation in the NiO-NP-treated tissue.

## Materials and Methods

### Plant Growth Conditions, Treatment Schedule, and Procurement of Nickel Oxide Nanoparticle

Seeds of *A. cepa* were grown according to reported protocols (Manna and Bandyopadhyay, 2017a). In brief, seeds of *A. cepa* (var. Nasik Red), bought from Suttons Seeds Pvt. Ltd., Kolkata, were germinated and seedlings were grown in the Experimental Garden, Department of Botany, University of Calcutta. Fresh, healthy bulbs, grown under controlled conditions, were put on a wet bed made of sand, which was twice sterilized and moistened with double-distilled water and maintained in a growth chamber at 23 ± 2°C in the dark for rooting.

Engineered NiO-NP was obtained from Sigma-Aldrich (St. Louis, United States) as nanopowder (product code 637130, molecular weight: 74.69, EC number: 215-215-7, PubChem Substance ID 24882831, <50 nm particle size (TEM), purity—99.8% trace metal basis). Healthy uniformly rooted bulbs were selected for treatment with various concentrations of NiO-NP suspension (10, 25, 50, 62.5, 125, 250, and 500 mg L^–1^) for 24 h.

Multiple sets of negative and positive controls were simultaneously maintained under controlled conditions. The negative control set consisted of bulbs treated with double-distilled water only, whereas seven different positive control sets were devised to cross-check experimental parameters: C_1_—0.4 mM ethyl methane sulfonate (EMS)/1% Triton X; C_2_—10 mM sodium pyruvate (a H_2_O_2_ scavenger); C_3_—100 μM cPTIO (a nitric oxide scavenger); C_4_—25 μM H_2_O_2_; C_5_—100 μM cPTIO + 10 mM sodium pyruvate; C_6_—125 mg L^–1^ NiO-NP + 100 μM cPTIO + 10 mM sodium pyruvate; C_7_—25 μM H_2_O_2_ + 125 mg L^–1^ NiO-NP + 100 μM cPTIO + 10 mM sodium pyruvate; and C_8_—25 μM H_2_O_2_ + 125 mg L^–1^ NiO-NP + 100 μM cPTIO + 0.4 mM EMS + 10 mM sodium pyruvate (Supplementary Table 1).

After the 24-h treatment, bulbs were harvested and the roots used for the subsequent assays.

### Internalization of FITC-Tagged Nanoparticles

Uncoated NiO-NP were tagged to fluorescein isothiocyanate (FITC) following the protocol of Xia et al. (2008). NiO-NP were suspended in DMF (dimethylformamide) through sonication, followed by the addition of APTS (aminopropyl triethoxysilane). The suspension was kept under continuous stirring for 24 h, followed by centrifugation at 5,000 × *g*. The supernatant was discarded, while the nanoparticles were further suspended in a DMF and FITC mixture, with continuous stirring for another 12 h. After that, the tagged nanoparticles were thoroughly washed in DMF till the supernatant did not fluoresce any more. The FITC-tagged nanoparticles were resuspended in deionized (DI) Milli-Q water and used in dose-dependent treatments to study internalization of the ENP.

Rooted bulbs were dipped in FITC-tagged NiO-NP suspensions for the requisite time periods. Root tips of onion bulbs were collected after treatment, thoroughly washed with DI water, hydrolyzed for 60 s in 1 N HCl, and squashed on clean grease-free slides (Manna and Bandyopadhyay, 2017b). The slides were studied and documented under a confocal laser scanning microscope (Olympus, Japan) (1 × CLSM 81) using the software version: FluoView FV1000, 350–470 filter for confirming internalization of FITC-tagged NiO-NP in root cells.

### Qualitative and Quantitative Determination of Cell Death and Wall Damage Through Evans Blue Staining

Qualitative estimation of cell death in the NiO-NP-treated root tissues against the control sets was performed using Evans blue dye (0.025% of Evans blue in 1 × PBS). At least 10 root tips from each set were immersed in the dye for 15 min and kept in the dark at room temperature, then washed repeatedly in PBS and observed with a simple microscope under 4 × objective (Zeiss-Primo Star). The Evans blue-stained root tips were immersed in 1 ml of 1% SDS buffer, crushed and centrifuged at room temperature, followed by measurement of color intensity at 600 nm (Vijayaraghavareddy et al., 2017) in a UV–VIS Spectrophotometer (Techcomp). Cell viability percentage was calculated as per the formula used in Manna and Bandyopadhyay (2017b).

### Qualitative Estimation of Lipid Peroxidation Through Schiff’s Staining

Lipid peroxidation as a result of NiO-NP treatment was determined according to Yin et al. (2010) with slight modifications. Fresh roots of both control and treated samples, at least 10 in number from each set, were treated with Schiff’s reagent for 15 min and washed thoroughly with 0.5% sulfite solution, then observed under a simple light microscope (Carl Zeiss-4 × objective) and photographed accordingly. Qualitative determination of color intensity was taken to be indicative of the extent of lipid peroxidation.

### Quantitative Determination of Gamma Amino Butyric Acid

Changes in gamma amino butyric acid (GABA) contents in NiO-NP-treated sets were assessed using the protocol of Zhang et al. (2014). One gram of root tissue was homogenized in 5 ml deionized water, vortexed for 30 min, and filtered using Whatman no. 1 filter paper. Then equal volumes of filtrate and 0.2 M borate buffer, along with an equal volume of 6% phenol and half volume of 9% sodium hypochlorite, were added, mixed thoroughly, and boiled for 10 min before immediately cooling in an ice bath. The blue color that developed was measured spectrophotometrically at 645 nm. The absolute value of GABA content was determined through a GABA standard curve (nmol g^–1^ fresh weight).

### Estimation of Nitric Oxide in Treated Tissues

#### Qualitative Detection of Nitric Oxide

NO was measured using the ubiquitous NO detecting fluorescent dye DAF-FM-DA (4-amino-5-methylamino-2′,7′-difluorofluorescein diacetate, Thermo Fisher Scientific) according to the protocol of McInnis et al. (2006) with minor modifications. Root tips were excised from the treated bulbs and immediately immersed in 1 × PBS for 15 min followed by transfer to 2 μM solution of DAF-FM-DA in 1 × PBS and incubation for 15 min. The root tips were then washed in PBS and viewed under the confocal laser scanning microscope (Olympus, Japan) (1 × CLSM 81) at excitation and emission of 495/515 nm. Images of the root tips showing bright green spots of NO localization were captured using the software version FluoView FV1000.

#### Quantitative Estimation of Nitric Oxide *in situ* (Griess Assay)

Freshly treated root tissues were crushed in phosphate-buffered saline in an ice bath and centrifuged at 4°C for 30 min, and the supernatant was used for further analyses. Since nitrite is the only stable component that can convert into NO, 1 U nitrate reductase (NR, Sigma) was added to the suspension which was then incubated for 30 min; 100 μl of supernatant from each set was then dispensed into a plate reader in triplicate, and an equal volume of Griess reagent [1% sulfanilamide in 5% phosphoric acid; and 0.1% N-(1-naphthyl)ethylenediamine dihydrochloride; Sigma-Aldrich] was added, before incubation for 15 min. The color reaction was assayed using a Bio-Rad iMark^TM^ microplate reader at 550 nm (Saha et al., 2004) and expressed as μg g^–1^ fresh weight.

### Quantitative Determination of NOS-like, NOX, and NR

Nitric oxide synthase-like activity was quantified in the root tissues of both treated and control *A. cepa* sets following the protocol of Murphy and Noack (1994) with necessary modifications. Five hundred milligrams of fresh tissue was crushed in a Tris homogenization buffer, containing 0.5 mM EDTA, 1 M leupeptin and pepstatin, 7 mM glutathione, and 0.2 mM PMSF, followed by centrifugation at 10,000 × *g*. The supernatant was recovered and centrifuged again at 80,000 × *g* for 30 min. Part of the supernatant was used to quantify total protein using the Bradford assay (Bradford, 1976) and the rest was mixed with Griess reagent [1% sulfanilamide in 5% phosphoric acid; and 0.1% N-(1-naphthyl)ethylenediamine dihydrochloride]. NR present in the supernatant reacted with available nitrite to give a bright purple azo dye that was measured at 546 nm and expressed as nmol min^–1^ ml^–1^.

NADPH-oxidase-like enzyme (NOX-like) was quantified in the experimental samples following the protocol of Libik-Konieczny et al. (2015). Fresh root tips were homogenized in protein extraction buffer containing 0.25 M sucrose, 10 mM Tris–HCl, 1 mM EDTA, and 2.5 mM DTT (dithiothreitol). The filtrate was centrifuged at 10,000 × *g* for 15 min at 4°C. The supernatant was separated and centrifuged at 80,000 × *g* (in ultracentrifuge, Thermo Scientific, Sorvall WX 80) for 30 min at 4°C. The pellet was collected and resuspended in Tris dilution buffer. Protein content was measured through the Bradford assay. A reaction mixture consisting of 1 M Tris buffer, 1 mM XTT, 1 mM NADPH, and membrane protein in the form of the resuspended pellet was prepared and measured at 490 nm. The activity was measured through the extinction coefficient of O_2_^–^ generation expressed as nmol min^–1^ ml^–1^.

NR was quantified following the protocol of Vega and Kamin (1977) with modifications. Initially, fresh root tips of the treated and untreated samples were homogenized in 50 mM Tris–HCl buffer, and the supernatant was used for further analyses. The reaction mixture consisted of 0.5 M Tris buffer, 2.5 mM sodium nitrite, and 3 mM methyl viologen to which the supernatant was added along with 0.025% of dithionite-sodium carbonate. The mixture was incubated for 15 min at 30°C and shaken well till the blue color disappeared. At this point, Griess reagent was added and absorbance was measured at 550 nm and expressed as nmol min^–1^ ml^–1^.

### Quantitative Determination of Hydrogen Peroxide and Proline

Quantification of H_2_O_2_ was done following the protocol of Velikova et al. (2000) with minor modifications. In short, 100 mg of fresh root tissue was homogenized in ice-cold 0.1% TCA (trichloroacetic acid) and centrifuged at 14,000 × *g* for 30 min. Following this, 50 μl of the supernatant along with the same volume of phosphate buffer was incubated with double the volume of 1 M potassium iodide (KI) solution. The resultant mix was read at 390 nm in a plate reader (Bio-Rad, iMark^TM^). The exact amount of H_2_O_2_ was calculated from a standard curve as nmol g^–1^ fresh weight.

Free proline content in the treated sets of *A. cepa* roots was estimated against the controls and detected following the protocol of Bates et al. (1973) with modifications; 100 mg of root tissue was homogenized in 3% sulfosalicylic acid and centrifuged at 10,000 × *g*. An equal volume of supernatant was reacted with freshly prepared acid–ninhydrin and glacial acetic acid at 100°C. The pinkish-red chromophore that developed was measured at 520 nm in a spectrophotometer (Techcomp). Quantification was done by following a standard curve of proline expressed as nmol g^–1^ fresh weight.

### Quantitative Ratio of Intracellular Potassium–Sodium Ions

Potassium:sodium ratio of all the experimental sets of *A. cepa* was determined through inductively coupled plasma-optical emission spectroscopy (ICP-OES) (Manna and Bandyopadhyay, 2017a) (Perkin Elmer Optima 5300 DV, United States) following a wet digestion protocol (Cramer et al., 1990). One gram of fresh root tissue was kept in 80% nitric acid for 10 days, followed by another round of acid digestion in which the partially digested tissue was heat digested in concentrated nitric acid and hydrofluoric acid followed by filtration and dilution.

### Quantification of Antioxidant Enzymes Using Biochemical Assays and Detection of mRNA Transcript Using Semiquantitative Reverse Transcriptase PCR

The important antioxidant enzymes, catalase (CAT), superoxide dismutase (SOD), and ascorbate peroxidase (APX), were quantified to understand their activities in both the treated samples and the control sets (negative control sets and the positive control sets). CAT and SOD were measured according to Manna and Bandyopadhyay (2017a), and APX was measured according to the protocol depicted by Das et al. (2018). One hundred milligrams of fresh root tissues were collected from all the sample sets, crushed in liquid nitrogen with 50 mM phosphate buffer (pH 7.4), and centrifuged at 1,000 × *g* for 15 min. The supernatant was collected and total protein was calculated using the Bradford reagent (Bradford, 1976) in a multiwell microplate reader (Bio-Rad, iMark^TM^) at 600 nm. Enzyme activities were measured using the individual coefficients of the enzymes spectrophotometrically (Technocomp) and each expressed as activity nmol min^–1^ ml^–1^.

Changes in the expression of transcripts of stress-responsive genes were studied using qRT-PCR. CAT, SOD, APX, and the two subunits of Rubisco activase (RCA) were chosen for the study following Thiruvengadam et al. (2015). Freshly treated roots from *A. cepa* were used to extract RNA following the manufacturers’ instructions (NucleoSpin RNA Plant kit, TaKaRa Bio, Cat 740949.50). First-strand cDNA synthesis followed by reverse transcriptase PCR was done using a single reaction by TaKaRa one-step RNA PCR Kit (AMV, Cat RR024B) in a single tube. PCR was carried out in a reaction mixture containing 1× one-step RNA PCR buffer, 5 mM MgCl_2_, 1 mM dNTP mixture, 0.8 units/μl RNase inhibitor, 0.1 units/μl AMV RTase XL, 0.1 units/μl AMV Optimized Taq, 0.4 μM forward and reverse primers, 1 μg of total RNA, and RNase-free double-distilled water. RT-PCR condition was set as 30 min at 50°C for reverse transcription, 2 min at 94°C for inactivation of RNase, followed by 30 cycles of 94°C for 30 s, annealing temperature for 30 s and 3 min at 72°C for an extension. An equal volume of PCR products was used for electrophoresis on 1.5% (w/v) ethidium bromide-stained gel. Gels were observed under UV light. Actin is used as reference gene for the present study. The gene-specific primers used in this study are listed in Supplementary Table 2.

### Statistical Analyses

All the experiments were carried out at least thrice to check the authenticity of the data.

Statistical analyses were carried out in the MINITAB environment (MINITAB ver. 18). Data were presented as mean ± standard error. The results were subjected to a one-way analysis of variance (ANOVA). The level of significance was established at *p* > 0.05 at every instance for all the biochemical assays, throughout the study. In case a significant interaction was found among the factors (species × concentrations of NiO-NP or that of various positive controls), such instances were compared by Tukey’s test and Dunnett’s test (Manna and Bandyopadhyay, 2017b).

## Results

### FITC-Tagged NiO-NP Helped Trace the Entry and Mobilization of ENPs Within Exposed Roots

**FIGURE 1:**
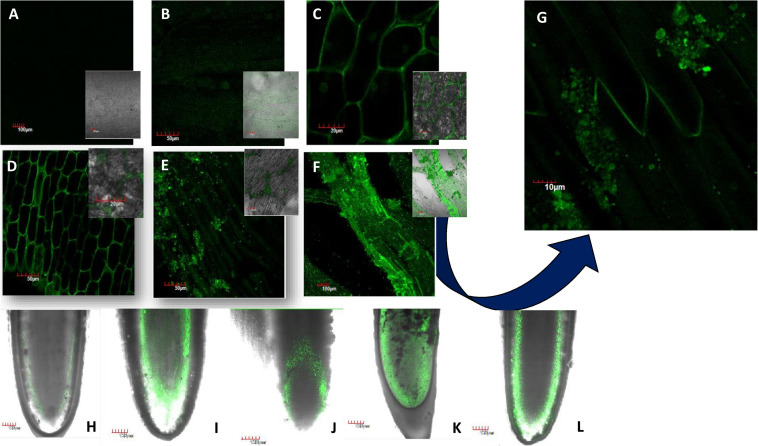
Translocation of FITC-tagged nickel oxide nanoparticles (NiO-NP) entering the tissue and adhering to it in a dose-dependent manner [**(A)** negative control, **(B)** tissue exposed to 10 mg L^–1^, **(C)** tissue exposed to 50 mg L^–1^, **(D)** tissue exposed to 125 mg L^–1^, **(E)** tissue exposed to 250 mg L^–1^, **(F)** tissue exposed to 500 mg L^–1^, **(G)** tissue exposed to 250 mg L^–1^—magnified view showing NiO-NP aggregates on the membrane surface; **(H–L)** vertical translocation of NiO-NP from the meristematic zone to the zone of elongation—**(H)** negative control, **(I)** 25 mg L^–1^, **(J)** 62.5 mg L^–1^, **(K)** 125 mg L^–1^, **(L)** 500 mg L^–1^].

Fluorescein isothiocyanate-tagged ENPs were internalized in *A. cepa* roots within 24 h of treatment with different concentrations of NiO-NP as seen in Figure 1. Figures 1A–G confirm the transit of NiO-NP into the cytoplasm of the epidermal cells and their entry through the nuclear membranes as well. With increased concentration of NiO-NP, higher fluorescence intensity could be detected in the cytoplasm of root tip cells, indicating toward a dose-dependent increment of tagged ENP entry. This corroborates our earlier findings where ICP-OES data had also shown a dose-dependent increase in NiO-NP content in the treated tissues (Manna and Bandyopadhyay, 2017a).

Figures 1H–L trace the gradual movement of FITC-tagged NiO-NP through root tissues, from epidermal cells to apical meristem cells at the tip, and then moving through the cortex along the zone of elongation, finally reaching the vascular tissues. While both the epidermis and cortical regions of exposed root tips were dotted with specks of FITC-doped NiO-NP in all concentrations (Figures 1A–G), it was only at higher concentrations that these were observed in the permanent tissues of the roots, too (Figures 1H–L). The presence of FITC-tagged NiO-NP in the vascular bundles alludes to the possibility of transmission of ENP to the aerial parts of exposed plants. Another interesting observation was that at higher doses, NiO-NP agglomerated to form clumps around the membranes of exposed epidermal cells, simulating their bulk counterparts as shown by the SEM images of affected root tips (Supplementary Figure 1).

### NiO-NP Treatment Caused Substantial Wall Damage, Lipid Peroxidation, and Perturbed K/Na Ratios in the Affected Cells

**FIGURE 2:**
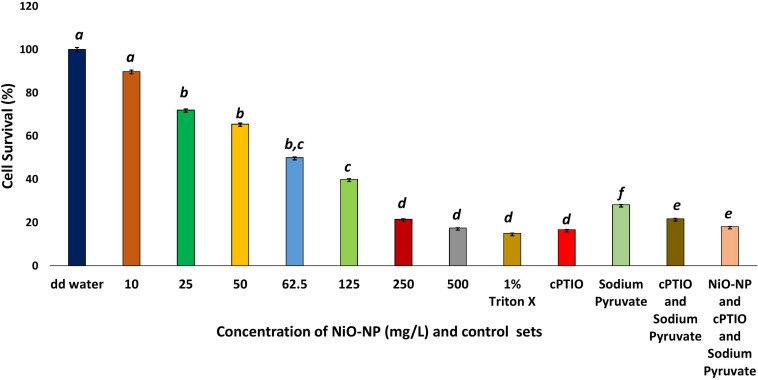
Cell survival percentage calculated through formazan formation. These letters denote statistical grouping after performing One-way ANOVA.

Evans blue staining of the treated and control sets of root tips showed a dramatic rise in cell wall damage and, thus, cell viability, coinciding with an increase in NiO-NP doses (Figure 2). As is known, dead cells with permeable membranes take up the dye which enters the protoplasm and stains it blue. Cumulative accumulation of the dye in damaged cells measured as the color intensity can be directly correlated to cell viability; more color accumulation indicated more membrane damage and, hence, less viable cells (Supplementary Figure 2). At the lowest dose of 10 mg L^–1^ NiO-NP, about 10% loss of cell viability was noted over the negative control, and at 50 mg L^–1^ NiO-NP, a 35.5% loss of cell viability was registered.

Interestingly, a similar proportion of loss in cell viability (∼82–85% loss over the negative control) was observed in the roots growing in the presence of 1% Triton X, as well as those growing in 100 μM cPTIO, a nitric oxide inhibitor, alone or in combination with 10 mM of the ROS scavenger sodium pyruvate (Figure 2) signifying NO plays a pivotal role in maintaining cellular viability.

The primary cause for cell membrane damage is lipid peroxidation that results in the formation of malondialdehyde as an aftermath of ROS-induced damage. Malondialdehyde formation can be macro-documented by Schiff’s reagent staining. NiO-NP-treated roots showed a sustained increase in the absorbance of the dye in a dose-dependent manner, indicating an increase in cell membrane damage with increasing NiO-NP doses (Supplementary Figure 3). Maximum lipid peroxidation was observed in the roots exposed to 250–500 mg L^–1^ of NiO-NP, in line with the observations from the cell viability assay.

**FIGURE 3:**
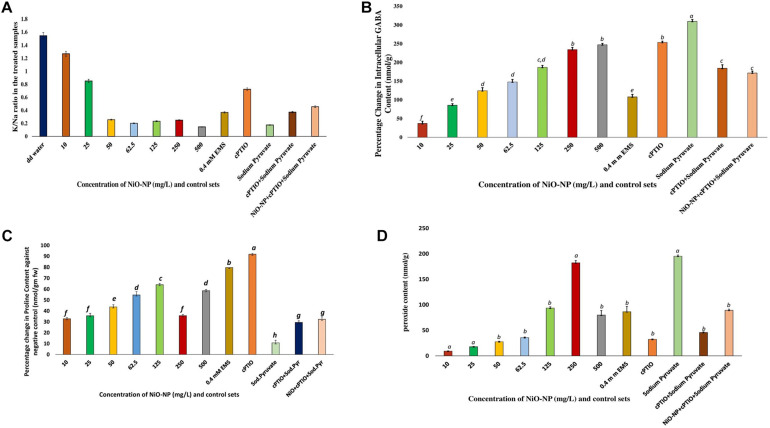
**(A)** Bar graphs showing the K/Na ratio in the exposed tissue against the negative control; **(B)** Bar graphs showing percentage change in gamma amino butyric acid content in the exposed tissue against the negative control (*p* < 0.05); **(C)** Bar graphs showing intracellular proline content in the exposed tissue against the negative control (*p* < 0.05); **(D)** Bar graphs showing intracellular hydrogen peroxide content in the exposed tissue against the negative control (*p* < 0.05). These letters denote statistical grouping after performing One-way ANOVA.

In the present study, a constant decrease in K/Na ratio was noted congruent to increasing NiO-NP dosages used, due to an increase of sodium ions in the cell with a concomitant decrease in the intracellular potassium pool in the affected tissues. In the negative control sets, this ratio was 1.544, while it reduced to 1.27 in the roots exposed to 10 mg L^–1^ NiO-NP and 0.8 upon exposure to 25 mg L^–1^ NiO-NP (Figure 3A). Subsequent decrease against the negative control coincided with a general deterioration in cellular well-being of the treated roots with increasing NiO-NP concentrations. All positive control sets also showed a considerable decrease in this ratio when compared with the negative control showing 0.4 mM EMS effectively reduced K/Na ratio and cPTIO blocked NO formation leading to decreased K/Na ratio, and blocking only H_2_O_2_ is not enough to reduce ROS-induced damages and it has a role in maintaining homeostasis.

### *A. cepa* Roots Show Rise in GABA, Proline, and Hydrogen Peroxide Concentrations on NiO-NP Treatment

Gamma amino butyric acid showed an upsurge in all the NiO-NP-treated tissues and in the positive control sets when compared with the roots growing as a negative control (DD water). In the negative control sets, GABA content was estimated to be 0.168 nmol g^–1^, and it increased to 0.231 nmol g^–1^ with a percentage increase of 85.7 in the roots exposed to 10 mg L^–1^ NiO-NP. In the roots exposed to median ranges of NiO-NP doses (50–125 mg L^–1^), GABA content increased by 124–187%. GABA contents in the roots treated with higher concentrations of NiO-NP (250–500 mg L^–1^) were comparable to the positive control sets. The percentage increase in the case of roots growing in 250 and 500 mg L^–1^ NiO-NP was 234 and 247, respectively, comparable to the roots treated with cPTIO and sodium pyruvate (Figure 3B), indicating that the absence of both H_2_O_2_ and NO is not beneficial for the treated tissue, showing their native roles in the maintenance of cellular metabolic activities.

Proline profiles of the treated and positive control sets were studied against the negative control set. As with GABA, proline content also showed a dose-dependent increase in the roots under NiO-NP treatment, up to a concentration of 125 mg L^–1^, as was the case in some of the positive control sets, too. While the percentage increase in proline content was 32.8 in the roots growing at 10 mg L^–1^ and 43.7 in those growing at 50 mg L^–1^ of NiO-NP treatment, at the median treatment concentration of 125 mg L^–1^ NiO-NP, an increase of 64% proline content over the negative control was noted. However, at higher concentrations of NiO-NP (250–500 mg L^–1^), proline content decreased substantially (58.58% decrease over the negative control at 500 mg L^–1^ NiO-NP). In the 0.4-mM EMS-treated set, a 91% increase of proline content over the negative control was observed (Figure 3C). However, in the other positive control sets, proline content reduced when compared with the negative control.

H_2_O_2_ content increased drastically, even in the roots exposed to the lowest dose of NiO-NP (10 mg L^–1^) when compared with the negative control. While the roots of negative control sets contained 0.023 nmol g^–1^ H_2_O_2_, in the 10 mg L^–1^ NiO-NP-treated roots, the total content rose to 0.045 nmol g^–1^. A concurrent increase in H_2_O_2_ content was noted in the subsequent higher concentrations of NiO-NP as well, with an almost 900% increase to that of the negative control observed from 62.5 mg L^–1^ onward. In the case of cPTIO-treated root tissues, H_2_O_2_ content was almost 1,900% more than the negative control, directly correlating a lack of NO with increased H_2_O_2_ production. Roots treated with 500 mg L^–1^ NiO-NP showed similar H_2_O_2_ content with that of the roots exposed to 0.4 mM EMS (Figure 3D).

### Nitric Oxide Shows Upsurge Upon Exposure of *Allium cepa* Roots to NiO-NP

**FIGURE 4:**
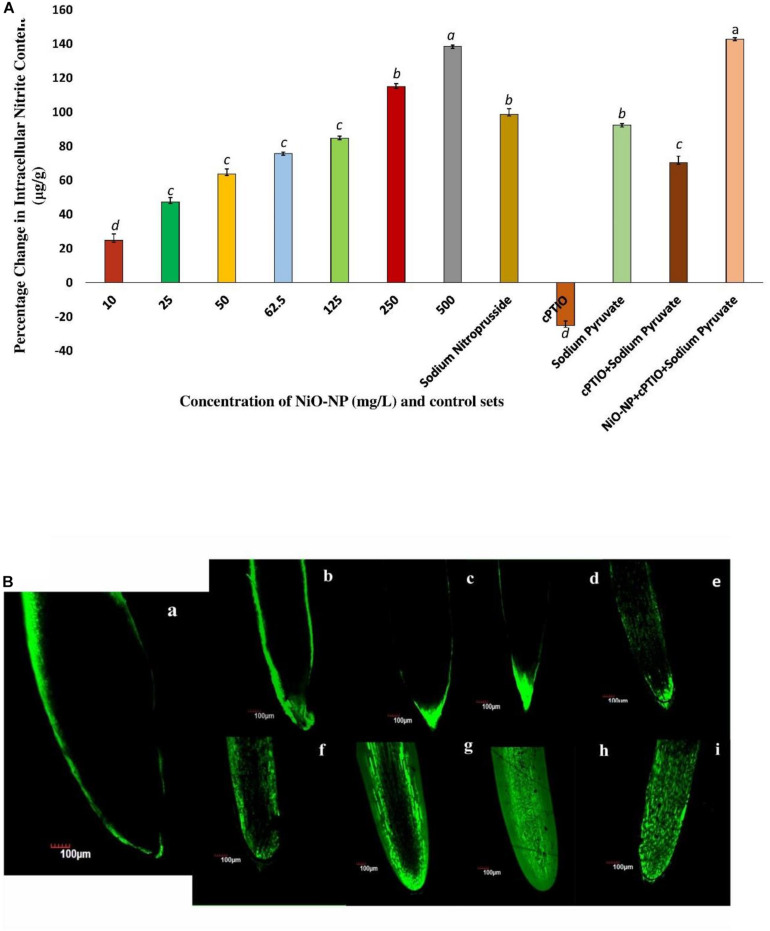
**(A)** Bar graphs showing intracellular nitric oxide content in the exposed tissue through Griess reagent (*p* < 0.05); **(B)** Root tips stained with DAF-FM-DA showing the extent of nitric oxide formation: **(a)**, negative control; **(b)**, tissue exposed to 10 mg L^–1^ NiO-NP; **(c)**, tissue exposed to 25 mg L^–1^ NiO-NP; **(d)**, tissue exposed to 50 mg L^–1^ NiO-NP; **(e)**, tissue exposed to 62.5 mg L^–1^ NiO-NP; **(f)**, tissue exposed to 125 mg L^–1^ NiO-NP; **(g)**, tissue exposed to 250 mg L^–1^ NiO-NP; **(h)**, tissue exposed to 500 mg L^–1^ NiO-NP; **(i)**, tissue exposed to 100 μM cPTIO + 125 mg L^–1^ NiO-NP + 10 mm sodium pyruvate. These letters denote statistical grouping after performing One-way ANOVA.

Griess assay is a sensitive analytical test where gaseous NO is quickly converted to nitrite form, for absolute and easy detection through color formation. In the present study, a concurrent increase in nitrite content was observed which was in tandem to qualitative data generated using DAF-FM-DA staining of experimental sets *via* confocal microscopy (Figures 4A,B). A basal level of NO was detected in the roots of the negative control sets, which surged upon the introduction of NiO-NP even at the lowest dose (10 mg L^–1^), showing an increase of 24.77% in NO content over the negative control. Exposure to 50 mg L^–1^ NiO-NP caused a 63.82% increase in NO content over the negative control, while around 85% increase was noted in the tissues growing at 125 mg L^–1^ NiO-NP. At higher doses (250–500 mg L^–1^) of NiO-NP, a further spike in NO content was noted (around 115 and 140% increase, respectively, over the negative control), which again was in concert with the confocal microscopic observations (Figure 4A).

*Allium cepa* roots of all the treated and control sets were studied through laser scanning confocal microscopy, after staining with DAF-FM-DA, a NO detector at the cellular level. An increase in color intensity directly dependent on the NiO-NP dose was observed, signifying the trend in increase in cellular NO levels under increasing ENP exposure. While a stable NO content was detected even in the negative control sets (Figure 4B), the introduction of a heavy metal nanoparticle was found to instigate a sudden upsurge in NO levels in the exposed tissues. From a slight rise in intracellular NO at the lowest dose of 10 mg L^–1^ NiO-NP (Figure 4A), a further increment in NiO-NP dose resulted in increased fluorescence of DAF-FM-DA. At the median concentrations of NiO-NP (50–125 mg L^–1^), NO content and fluorescence increased prominently, and at higher concentrations (250–500 mg L^–1^), as well as in the positive control sets, characteristically enhanced fluorescence was observed, pointing to the presence of excessive NO in treated tissues (Figure 4Bi).

### Quantification of NOS-like, NOX, and NR Enzymes Confirm Augmented NO Production in the NiO-NP-Affected Tissue

**FIGURE 5:**
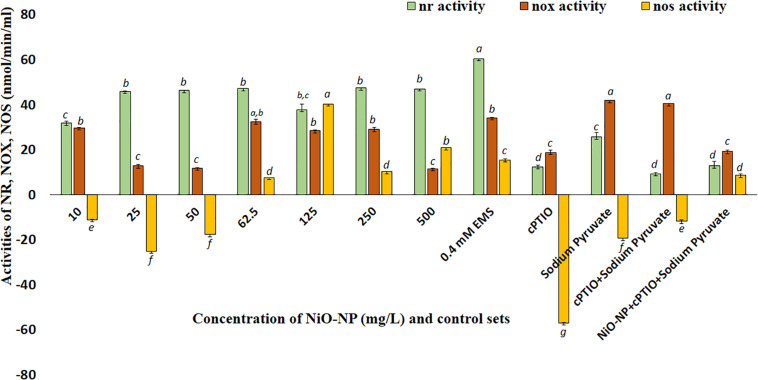
Bar graphs showing nitric reductase, NADPH oxidase, and nitric oxide synthase activity in the exposed tissue against the negative control (*p* < 0.05). These letters denote statistical grouping after performing One-way ANOVA.

Nitric oxide synthase-like proteins and NR are among the few known enzymes responsible for NO production in plants, and thus, their activities were quantified to understand fluctuations in NO production. NOX-like activity increased consequent with an increase in cellular ROS in the present study, as was also reported earlier (Yadu et al., 2018). NR and NOS-like activities both showed dose-dependent increase in the treated tissues when compared with the negative control. NR activity showed an upturn in roots exposed to even the lowest dose of 10 mg L^–1^ NiO-NP used (30% increase over the negative control). With subsequent increments in NiO-NP concentrations, there was a gradual increase in NR activity with a 45–47% increase over the negative control across all the sets (Figure 5). In the case of NOS-like activity, however, a decrease was noted in the roots exposed to the lower doses of NiO-NP (10–50 mg L^–1^), which was around 40% less than that of the negative control. A further increase in NiO-NP doses increased NOS-like activity, with a 40% augmented activity observed at 125 mg L^–1^ NiO-NP (Figure 5). NOX showed increased activity in the NiO-NP-treated sets, too, though the increase was not dose-dependent. There was around 30% increase in the roots growing at 10 mg L^–1^ NiO-NP, whereas a 12% increase was noted in the tissues exposed at 50 mg L^–1^ NiO-NP. At higher concentrations, there was only a nominal increase in NOS-like activity (Figure 5).

### Biochemical Assays and Semiquantitative RT-PCR Confirmed Increased Activity and Upregulation of Important Antioxidant Enzymes and Genes in NiO-NP-Exposed Roots

**FIGURE 6:**
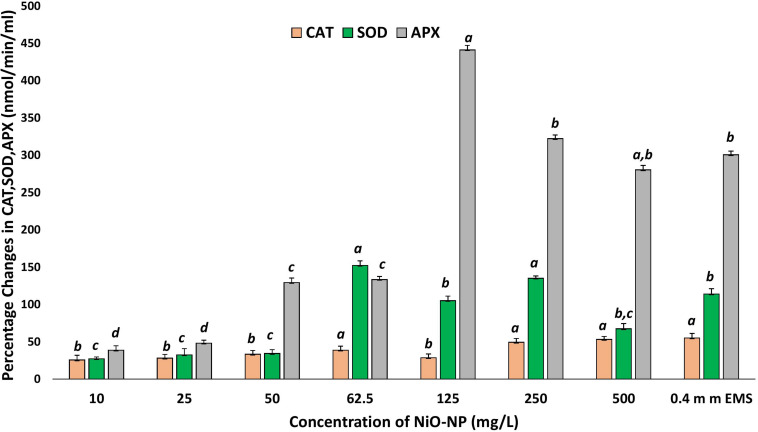
Effect of increasing NiO-NP concentration on catalase and SOD activity and on APX activity, expressed as percentage change (*p* < 0.05). These letters denote statistical grouping after performing One-way ANOVA.

Nickel oxide nanoparticles can cause perturbation in antioxidant activities as a direct outcome of oxidative stress. CAT, SOD, and APX contents were measured to study the extent of oxidative damage induced by NiO-NP in the treated samples. CAT showed a significant increase of 25% at 10 mg L^–1^ NiO-NP treatment over the untreated samples. As NiO-NP concentration increased further, CAT content increased to 38% over the negative control at 62.5 mg L^–1^ NiO-NP dose and 49% increase over the untreated sets at 250 mg L^–1^ NiO-NP concentration (Figure 6). SOD also followed a similar trend, where an initial increase of 28% activity over the negative control was noted at 10 mg L^–1^ followed by a significant increase as the dose of NiO-NP increased, and a 150% increase over the negative control was witnessed at 62.5 mg L^–1^ NiO-NP concentration (Figure 6). APX activity increased notably in the treated sets against the negative control at all the concentrations of NiO-NP. There was an increase of 38% APX activity over basal control levels even at the lowest dose of NiO-NP (10 mg L^–1^). APX activity continued to increase across all the concentrations of NiO-NP. The highest APX activity was seen at 125 mg L^–1^ NiO-NP dose (440% increase) over the untreated control sets (Figure 6).

**FIGURE 7:**
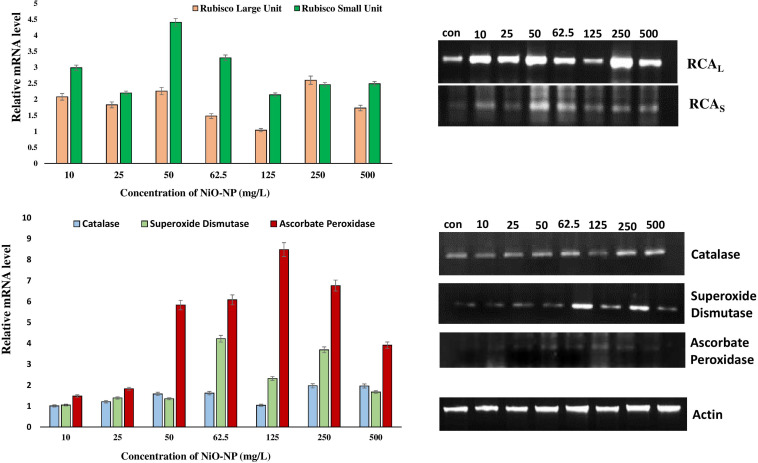
Representative gels showing the activities of different antioxidant genes and RCA subunits in *Allium cepa* control roots and NiO-NP-treated roots with their relative mRNA levels with the reference gene.

Cytotoxic aspects of NiO-NP have already been speculated using various biochemical assays, but the exact molecular mechanism of antioxidant profile perturbation was investigated using qRT-PCR. The expressions of crucial antioxidant genes, namely, CAT, SOD, and APX, and the RCA (large and small units) were studied in treated and untreated sets, and they showed upregulation in all the treated sets when compared with the negative control. CAT expression showed a maximum of 1.97-fold increase at the highest concentration of NiO-NP (500 mg L^–1^), while a 1.5-fold increase was seen in the roots exposed to median concentrations (125–250 mg L^–1^). SOD transcripts also showed an average 2-fold increase in expression at the median and higher concentrations, with the highest 4.22-fold increase observed at 62.5 mg L^–1^ NiO-NP concentration. The APX gene showed the highest expression among all the genes studied upon NiO-NP exposure. An 8.5-fold increase was documented in the tissues growing at 125 mg L^–1^ NiO-NP, while a 5.8-fold increase was seen at 50 mg L^–1^ NiO-NP (Figure 7). Both the RCA large and small transcripts showed median upregulation at all the NiO-NP concentrations. While the expression of the small subunit increased by 2–4-folds across all the concentrations of NiO-NP tested, the large subunit showed an average 1.5-fold upregulation (Figure 7). Actin was used as a reference gene that showed constant expression in all the samples (control sets and treated ones), and the quantitative level of actin is shown in Supplementary Figure 4.

## Discussion

Increased industrial uses of NiO-NP and its associated introduction into the natural environment in recent decades have drawn scientific attention to the specter of its harmful effects on plants as well as in humans (Chung et al., 2019; Cambre et al., 2020). Nickel is already categorized as a potent carcinogen by the WHO, and when it became known that the NiO-NP can mimic the activities of their bulk counterparts especially at higher concentrations, it became imperative that the exact mechanism of NiO-NP-induced damage to biological systems be studied in detail. Even though there are a few reports of NiO-NP-induced toxicity in plants underlining its dose-dependent toxicity, the whole gamut of physicochemical perturbations is yet not elucidated. One of the huge gaps in information that clouds our understanding is the dynamics of ROS and NO relation. Both ROS and NO are important participants in the normal cellular signal transduction that, at low concentrations, help in preserving cellular homeostasis (Romero-Puertas and Sandalio, 2016). Their integrative role in plants, from development to stress response, has been a topic of active research (Asgher et al., 2017). In this manuscript, the authors have ventured to augment existing information on how ROS and NO act in tandem in case of the advent of an engineered nanoparticle, and how an increase in NO can trigger ROS overproduction inadvertently causing physicochemical upheavals due to rampant oxidative damage. It was during this work that it became obvious that NiO-NP-induced toxicity incremented in a dose-dependent manner, leading to an overproduction of both ROS and NO. These two crucial components of the stress management cascade work initially in tandem, but at higher doses of NiO-NP (250–500 mg L^–1^), they behave antagonistically, leading to acute cellular damages.

The present experimental parameters accommodated a wide range of NiO-NP concentrations (10–500 mg L^–1^) keeping in mind their natural distribution and concentrations of Ni encountered in nature, which could go up to 1,600 mg kg^–1^ in coalfields^3^. It was noteworthy that *A. cepa*, the chosen plant model, did not show documentable disruption in cellular functions at lower doses of NiO-NP (below 10 mg L^–1^) and almost 100% cell death at concentrations higher than 500 mg L^–1^ after an exposure of 24 h. *A. cepa* is a popular choice for the detection of cytotoxicity and genotoxicity of any xenobiotic compound as endorsed by the various international organizations of repute like UNEP, USEPA, and OECD (Boros and Ostafe, 2020)^4^. It is easily available and fast-growing and shows uniform growth and comparable effects that can be extrapolated to mammalian systems. Also, parts of *A. cepa* are consumed raw, opening the possibility of NiO-NP introgression into the food web and its biomagnification. The authors have shown earlier that *A. cepa* roots take up NiO-NP readily, most probably *via* the plasmodesmata, in a dose-dependent manner (Manna and Bandyopadhyay, 2017b).

In the present work, FITC-tagged NiO-NP were utilized to unravel the dynamics of its internalization within tissues and cells of *A. cepa* roots using a confocal laser scanning microscope. FITC, possibly the most widely known fluorescent tracer used in biological sciences, has a reactive isothiocyanate which can be tagged to amine and sulfhydryl groups of different biomolecular moieties. FITC also finds widespread use as a tag for nanoparticles to check and record their entry into biological systems and localize them within cells and tissues, aiding biomedical studies (Veeranarayanan et al., 2012; Huang et al., 2018). In plants, FITC tagging of chitosan (Chandra et al., 2015) and zein nanoparticles (Ristroph et al., 2017) is reported. Other authors have also documented the entry of FITC-tagged zinc and cerium metallic nanoparticles into the cells (Xia et al., 2008). In the present study, it was observed that hydroponically administered FITC-tagged NiO-NP breached the root epidermis and were translocated to the meristematic zone, and then to the zone of elongation and differentiation, in a progressively dose-dependent manner (Figure 1). These NP entered individual cells and were seen scattered in the cytoplasm or accumulated around the nuclear membrane (Figure 1). From these pieces of evidence, we can deduce that the *modus operandi* of NiO-NP-induced toxicity rests on their ability to gain entry into the cells, causing damage to important organelles like the nucleus, peroxisomes, or mitochondria, which showed hindered functioning, similar to those reported in cases of NiO-NP in tomato seedlings by Faisal et al. (2013) and of CuO-NP on soybean (Yusefi-Tanha et al., 2020) earlier.

An increase in NiO-NP dosage brought about a steady deterioration of cellular membrane integrity, as was reported earlier (Manna and Bandyopadhyay, 2017b). Earlier reports have indicated that capricious numbers of aggregated NiO-NP of various sizes and conformations clustered over the epidermal cell membranes, abrading them, and caused substantial damage to their surfaces, before gaining entry into the cells (Ates et al., 2016). Such damage to the membrane surfaces led to an immediate increase in cellular ROS (Sohaebuddin et al., 2010; Shang et al., 2014). Uncontrolled ROS upsurge induced malfunction of the antioxidant profiles and indirectly compromised integrity and, thus, survival in the treated tissues, as documented by Evans blue staining of root tips in the present study. Evans blue, an azo dye, can penetrate into damaged and ruptured membranes more easily than healthy membranes (Vijayaraghavareddy et al., 2017). Hence, normal cells do not take up the blue color, while those with membrane damage do. The intensity of the color reaction can be studied spectrophotometrically to ascertain the quantum of membrane damage in the treated tissues. Increasing color intensities in the root tissues treated with NiO-NP showed prominent loss of cell viability with rising concentration of the ENP. Lipid peroxidation, an early sign of membrane damage, occurs when unstable membrane lipids form malondialdehyde that can react with Schiff’s reagent on the aldehyde group to produce a colored output (Awasthi et al., 2018). Interestingly, exposure to bulk nickel does not produce significant lipid peroxidation in affected tissue (unpublished data). However, *A. cepa* roots exposed to NiO-NP showed significant lipid peroxidation that was confirmed biochemically by TBARS assay earlier (Manna and Bandyopadhyay, 2017b). In the present work, also color reaction with Schiff’s reagent observed in the affected tissues increased with increasing doses of NiO-NP exposure (Supplementary Figure 3). It is known that during initial treatment with heavy metals, plants undergo dehydration (Singh et al., 2016), similar to osmotic stress (Osakabe et al., 2013). In a tissue having depleted potassium and augmented sodium ion contents, the ionic homeostasis becomes unhinged leading to cellular shutdown (Osakabe et al., 2013). Hence, the K/Na ratio was studied in a bid to find out possible changes in the ionic balance of the affected tissues. A marked dose-dependent decrease in K/Na ratio was observed in exposed root tissues upon NiO-NP treatment, similar to those seen in plants affected with dehydration and salinity stress confirming this ENP’s ability to damage cellular integrity and perturb ionic balance of affected cells. Thus, NiO-NP damages cell integrity leading to a cascade of physicochemical perturbations that induce stress in the exposed root tissues of *A. cepa*.

The increase in ROS content of tissues exposed to NiO-NP in the present experiment, especially H_2_O_2_, confirmed the trend of a dose-dependent upsurge in reactive species, as seen in earlier reports as well (Manna and Bandyopadhyay, 2017a,b). ROS acts as a double-edged sword that has immense importance in signal transduction and stress management. Exposure of plants to metals induces oxidative challenges through pathways specific to a particular metal (Rizwan et al., 2017), often culminating in a mismatch between production and neutralization of ROS, most notably H_2_O_2_, hydroxyl ion, and superoxides (Cuypers et al., 2016). H_2_O_2_ is a selectively reactive, uncharged non-radical, having both oxidizing and reducing properties, making it crucial for energy-efficient stress mitigation (Bienert and Chaumont, 2014; Das and Roychoudhury, 2014). The mitochondria, despite being the primary location of H_2_O_2_ generation, suffer maximum damage from its excess in the cell. It is noteworthy that nickel, a non-redox active metal, can indirectly lead to an increase in intracellular ROS levels in exposed tissues, either by inhibiting specific enzymes by blocking their binding sites, or by diminishing cellular GSH pool, or affecting NOX, upending the cellular antioxidant profiles, thereby creating a ROS furor as seen in the present experimental system (Valko et al., 2016).

Coincidentally available NO also showed a marked change in the NiO-NP-treated tissue against the control sets. There was a rise in NO, even at the lowest dose of NiO-NP, followed by a marginally stalled increase in the treated tissues observed around the median dose of 125 mg L^–1^ NiO-NP, followed by a burst of NO production at the higher doses (Figure 4). The intricate interplay of various NO production mechanisms in the affected tissues, either independently or in conjunction with augmented ROS, can cause a spike in NO accumulation (Romero-Puertas and Sandalio, 2016). As mentioned earlier, NR, an important molybdenum-containing multiredox enzyme, is crucial for both nitrogen assimilation in plants and for the reductive pathway of NO production, thus maintaining NO homeostasis. NR effectuates the transfer of electrons from NAD(P)H to downstream substrates, producing NO in the process, which also makes it a major enzymatic producer of NO in plants (Chamizo-Ampudia et al., 2017). Another important mode of NO production is through the oxidative pathway, where NOS catalyzes the production of NO in animals, though the exact homomers have not yet been found in plants so far. Scientists have long debated about the presence of NOS-like protein in plants (Astier et al., 2018) and have suggested that these are protein complexes working in coordination with subunits of arginine, NADPH, and NOS co-factors. Though orthologous genes of NOS similar to animals were not detected in plants, numerous reports depicting the biochemical presence of L-arginine-dependent NOS-like protein complexes have been documented that perform a similar function (Corpas and Barroso, 2018). Recent reports suggest a lateral rise in NOS-like activity with abiotic stress factors (Asgher et al., 2017), similar to the observations documented by the authors in the present study. Studies have shown that it is NR that produces a bulk of NO in a plant from nitrate reservoirs and controls the first rate-limiting step and even producing NO under high nitrite accumulation or in acidic/anoxic conditions; however, the turnover rate for NO formation following this pathway is quite low. The reactivity of NOS-like enzymes, on the other hand, is affected by many individual co-enzymes and co-factors which constitute its active form. Hence, a consequential increase in NR becomes more prominent than NOS-like for NO production during conditions of stress (Astier et al., 2018). NR activity in exposed roots showed a dose-dependent linear rise in all the concentrations of NiO-NP used, with an initial rise of ∼30% at the lowest dose of 10 mg L^–1^ NiO-NP, followed by ∼47% increase at 250 mg L^–1^ NiO-NP and 80% increase at the highest dose of 500 mg L^–1^ NiO-NP. Thus, it becomes obvious that the NiO-NP-treated tissue implemented the reductive pathway as the major source of intracellular NO generation. There is no evidence of a direct correlation between NR and NOS-like activity in plant systems so far, but *in vivo* S-nitrosylation loop inhibits NO formation by NR, and also, NR is competitively inactivated by higher cellular nitrate concentration. Therefore, the presence of alternate NO-forming mechanisms is evidently present in every plant cell, the most eligible candidate for such being the NOS-like complexes (Fu et al., 2018). In the present study, there was an initial fall (11% depletion) in NOS-like activity in *A. cepa* root tips exposed to the lowest dose of NiO-NP (10 mg L^–1^) against the negative control, followed by a 17% fall in such activity in the roots treated with 50 mg L^–1^ NiO-NP. Interestingly, the roots exposed to NiO-NP ranging between 62.5 mg L^–1^ and above showed a steady rise in NOS-like activity with as much as a 40% rise at 125 mg L^–1^ NiO-NP concerning the negative control and 20% increase in activity at the highest dose of NiO-NP (500 mg L^–1^). Thus, NOS-like activity showed a non-linear correlation with NiO-NP dose among the treated samples, and these values are not significantly different among themselves in many instances. The present dataset corroborates that NOS-like activity is controlled by multiple factors, and perturbations in Ca^2+^ and/or NADPH that is inevitable in NiO-NP-affected tissue can also impact NOS-like activity (Faisal et al., 2013). In fact, detailed work by the present group has found increased cytosolic Ca^2+^ in the treated tissue, consequent to severe damages incurred by the chloroplast and mitochondria at toxic concentrations of NiO-NP (unpublished), alluding to such a possibility. Few reports that document the rise in both NOS-like and NR activity in higher plants subjected to abiotic stressors include the study of maize under dehydration stress that showed a rise in NOS-like activity on exogenous SNP (NO donor) application (Hao et al., 2008). In *Hibiscus*, aluminum treatment inhibited NOS-like activity but did not affect NR activity (Tian et al., 2007), while a rise in NR alleviated Al-induced damages in soybean and wheat (Wang et al., 2017; Sun et al., 2018). Cadmium exposure increased NR activity in alfalfa and barley (Lentini et al., 2018; Yang et al., 2019), which was similar to the observations recorded by the present authors in the case of NiO-NP stress on *A. cepa*. Our findings have indicated a dose-dependent increase in NR activity with rising NiO-NP concentrations and an accompanying increase in NO, thus affirming that NR acts in a linear manner using the cellular nitrate reservoir available to produce NO, which is in contrast to NOS-like activity which depends on various other intrinsic factors as well.

Exposure to increasing concentrations of NiO-NP incited an upsurge in NOX activity, which was concomitant with the higher levels of intracellular NO and was confirmed by the rise of H_2_O_2_ incidence, as evident even at the lowest ENP concentration used on *A. cepa* in the present study. The most prevalent enzymatic ROS producers in plants, NOX, are deemed as respiratory burst oxidase homologs (RBOHs). Implicated in several aspects of normal plant growth and development, these are also involved in maintaining the cellular balance of ROS and RNS (Chu-Puga et al., 2019). Increment in intracellular NO is known to increase NOX production since inflation of cellular H_2_O_2_ leads to an active breakdown of NADPH. This in turn augments NOX activity in the plasma membrane, which is primarily responsible for superoxide production in the apoplast of plants (Podgórska et al., 2017). Intrusions of xenobiotic agents are known to trigger NOX activity contributing to an oxidative burst. In the present study, an increase in NOX activity (∼30% more than negative control) was observed in *A. cepa* roots treated with 10 mg L^–1^ NiO-NP. Roots exposed to higher concentrations of NiO-NP showed a steady increase in NOX content, though not linear, with the highest increase documented at the dose of 500 mg L^–1^ NiO-NP (Figure 5). Our data corroborated recent reports of a dose-dependent increase in NOX activity in plants due to increased iron exposure (Yadu et al., 2018). A similar increase in NOX activity was observed upon cadmium and copper treatment in *Arabidopsis thaliana* roots (Remans et al., 2010) and Pb stress in the roots of *Medicago truncatula* (Zhang et al., 2019). Selenium-induced phytotoxicity in *Brassica rapa* also showed NOX-dependent ROS induction (Chen et al., 2014). Heyno et al. (2008) reported that treatment with heavy metals like cadmium induced ROS from the plasma membrane and not from the mitochondria. Similarly, the present work also confirmed increased plasma membrane was linked to NOX activity. There are confirmatory reports that, as a response to metal toxicity, cytosolic Ca^2+^ levels increased proportionately, activating and increasing NOX activity as well (Sosan et al., 2016). This in turn regulated cytosolic ROS generation through peroxidases (Heyno et al., 2008). Such concomitant steady increase in cytosolic Ca^2+^ levels with ROS increase was also recorded in the NiO-NP-treated sets of *A. cepa* (unpublished).

Intracellular levels of GABA and proline, both deemed to be universal markers of stress, were escalated with increased NiO-NP exposure in plants. GABA is an important non-protein amino acid useful in plant signaling, much like its role in the neurotransmission of animals (Ramos-Ruiz et al., 2019). It has an undeniable yet complex relationship with the ROS and RNS pools that has implications in increasing plant resilience to stress (Bor and Turkan, 2019). During oxidative stress response when intracellular ROS and related antioxidant levels inflate in a system, there is an impromptu rise in GABA levels as well, though whether as a cause or as its effect is yet debated. As the exogenous application of GABA reportedly induced antioxidant activity (Song et al., 2010), in the present work also, the authors have observed a marked increase in GABA content on NiO-NP exposure (Figure 3B), concomitant with a spike in ROS content and augmented antioxidant profiles. An increase of ∼37% GABA activity was observed in the roots exposed to the lowest dose of 10 mg L^–1^ NiO-NP, which increased to ∼190% at 125 mg L^–1^ of NiO-NP. A further dose-dependent increase in GABA activity was registered in the higher NiO-NP concentrations, too. Roots exposed to sodium pyruvate, a H_2_O_2_ inducer, also showed a significant increment in GABA content (∼110%) over the negative control. Since GABA is primarily synthesized by polyamines, indirectly dependent on the nitrite pool, it was not surprising that roots exposed to cPTIO, a NO blocker, did not show a significant enhancement of GABA content over the negative control. A major portion of the GABA shunt is located in the mitochondria (Bor and Turkan, 2019), which is also the epicenter of abiotic stress mitigation and signaling; as such, any disturbance of this organelle due to metal exposure can cause multiple physicochemical malfunctions. It has already been reported that NiO-NP exposure damages the mitochondria *via* perturbation of antioxidant activities, which could be correlated with possible alterations of the GABA shunt, too. Many groups in recent times have shown increased GABA content in various plants under heavy metal treatments. In *Nicotiana tabacum*, treatment with zinc increased the endogenous GABA levels which in turn augmented the antioxidant enzymatic activities of those plants (Daş et al., 2016). Wheat subjected to arsenic stress also showed higher levels of GABA content in the treated roots and shoots (Sil et al., 2018). Proline, another non-protein amino acid and marker of stress response in plants, is known to increase considerably with any exigencies, including metal-effected stress. In this work, a marked increase in proline content was recorded even at the lowest dose of NiO-NP against the negative control, with a linear dose-dependent increase in proline content in all the higher concentrations of NiO-NP, as also reported in many other plants exposed to other metallic nanoparticles. Barley plants treated with titanium oxide nanoparticles showed higher proline content than untreated control plants (Doğaroğlu and Köleli, 2017). Another heavy metal nanoparticle, copper oxide nanoparticles, also induced increased proline content in treated rice plants (Da Costa and Sharma, 2016). All these results were in tandem with the observations documented by the authors in the present study.

Antioxidant enzymes like CAT, SOD, and APX form the backbone of the stress mitigation system in a plant cell, responding minutely to any incoming stress signal and often leads to altered activities (Hasanuzzaman et al., 2019). In the present work, CAT, SOD, and APX showed significant increases following dose dependency of NiO-NP. These findings are in tandem to the previous reports available for NiO-NP-induced phytotoxicity. In fact, 24 h of uninterrupted NiO-NP treatment caused permanent damages to the framework of the cell as shown by decreasing cell survivability indices. Uninterrupted ROS generation, as shown by increased H_2_O_2_ accumulation, followed by excess NO generation led to multiple damages altering the biochemical framework of the cell and an imbalance in the activities of vital antioxidants (enzymes, metabolites), which also caused marked increase in proline and GABA levels along with changes in NO concentrations. A similar sequence of events was also observed when plants combat oxidative burst mechanism by a number of authors (Manna and Bandyopadhyay, 2017a; unpublished, Faisal et al., 2013; Oukarroum et al., 2015, 2017).

Semiquantitative reverse transcriptase polymerase chain reaction is especially convenient for the study of biomarker genes that are crucial for abiotic stress amelioration and survival (Qian et al., 2013). CAT, SOD, and APX, the chief players in the endogenous antioxidant systems of a plant cell, are instrumental in managing excess H_2_O_2_ and superoxides produced owing to oxidative burst linked to metabolic upheavals. Metallic nanoparticles often act as non-competitive inhibitors for several essential enzymes, like that of –SH groups of cysteine residues, forming covalent bonds and affecting folding during tertiary structure formation. This misfolding alters active site conformation, thus hindering the efficiency of many vital enzymes (Adeyemi and Whiteley, 2013) and affecting the normal metabolism of the affected cells. Enzymatic assay of CAT activity showed an upsurge in NiO-NP-treated roots against untreated sets (Manna and Bandyopadhyay, 2017b), as it participates in breaking down excess H_2_O_2_. Higher transcript levels of CAT in the present study affirm the previous report, proving evidence for the upregulation of CAT gene with increasing NiO-NP concentrations. APX, which not only helps detoxify excess H_2_O_2_ but also uses H_2_O_2_ for signaling and regulating the feedback loop of the GSH cycle (Singh et al., 2016), also showed increased transcript levels when compared with untreated controls. Higher transcript values of APX coincided with the general excess of H_2_O_2_ in the affected tissue. SOD also showed increased activity in the previous work reported by the authors detected through enzymatic assay, and the present data confirmed that SOD is the primary enzyme for dismantling the highly reactive superoxides. Thus, higher transcript levels maintained in tandem to oxidative burst indicate the intricate role SOD plays among stress-mitigating enzymes. All the concentrations of NiO-NP induced a general increase in activities of these antioxidants, except at 125 mg L^–1^, where a marked decrease in most of these enzyme activities was observed. This may be because this concentration was probably most toxic under specific biotic interactions for this ENP in this particular study, beyond which the formation of bigger clumps and their dissociation constantly alter the stoichiometry of individual NiO-NP units (Manna and Bandyopadhyay, 2017a,b) and prevent their entry into the tissue as well. Consistent results in many other heavy metals, like lead, cadmium, and arsenic, where antioxidant profiles have shown considerable upregulation in the treated plants (He et al., 2004; Bharwana et al., 2013; Lou et al., 2017), confirmed that antioxidant enzymes helped nullify the aftermath of metallic stress. Hence, it was deduced that NiO-NP caused major oxidative stress in affected plants and upregulation of antioxidant enzymes constituted a major form of defense responses. Rubisco (RuBP) is the primary enzyme required for carbon fixation in plants. It consists of eight subunits each of nucleus-encoded small units and chloroplast-encoded large units and is designated as a potential housekeeping gene, necessary for the well-being of any plant (Siedlecka et al., 1998). RCA is necessary for the formation of RuBP to maintain optimum carbon fixation, but it has been shown that abiotic stress signals necessitate its upregulation (Demirevska-Kepova et al., 2004). RCA activity is known to be adversely affected consequent to heavy metal intrusion (Khairy et al., 2016). Therefore, RCA is an important candidate in studying the extent of damage due to oxidative stress. In the present work, however, we have tried to interpret the effect of a heavy metal nanoparticle on its subunits, trying to understand whether they were being upregulated individually or in tandem. It is known that the two domains of ATP-dependent structures of RCA encode chloroplast proteins, with the large subunit being more sensitive to abiotic stress than the smaller one (Chen et al., 2015). RuBP is affected quite harshly during oxidative stress, marking a lower transcriptomic presence (Cohen et al., 2005). Antioxidants like GSH play an important role in maintaining RuBP levels in case of metabolic inconsistencies (Son et al., 2014), whereas APX is largely responsible for its proper functioning under optimum conditions, exerting an effect over a crucial rate-limiting step (Liu et al., 2008). In the case of oxidative stress in the form of NiO-NP intrusion, GSH levels deplete, while APX is negatively affected (unpublished). The interrelationship of NO and GSH is quite interesting though complex, and they form GSNO which could inhibit NR-related NO synthesis. NO, in turn, can react with the heme group, thus potentially inhibiting APX activity. In fact, NO affected all the enzymes of the Asa–GSH cycle, though report of its inhibition of APX has not been confirmed yet (Romero-Puertas and Sandalio, 2016). This explains the high expression level of APX in the treated tissue observed in the present study. Proline, the ubiquitous marker of ROS imbalance, when found in excess similar to that reported by the authors, is also known to lead to dissociation of RuBP subunits (Sivakumar et al., 2001). Demirevska-Kepova et al. (2004) reported a minor decrease in the activity of subunits of RCA in tomato treated with cadmium. Interestingly, in the present study, activities of both the subunits of RCA increased on NiO-NP exposure (Figure 7). According to Chen et al. (2015), such an event can lead to buffering of carbon fixation in a toxic environment. In case of the larger subunit, an increase in its activity in the NiO-NP-treated sets was only marginal and not statistically significant in all the concentrations. However, the activity of the smaller subunits showed a 2-fold increase in all the NiO-NP doses, making the change more relevant and contributing to an overall increase of RCA activity. Such an increase in RCA activity was observed in *Populus nigra* leaves on cadmium stress (Lomaglio et al., 2015). Higher intracellular NO content restored RCA activity in a stressed tissue (Khairy et al., 2016), and a similar trend was observed here where both intracellular NO content and RCA activity showed inflation.

**FIGURE 8:**
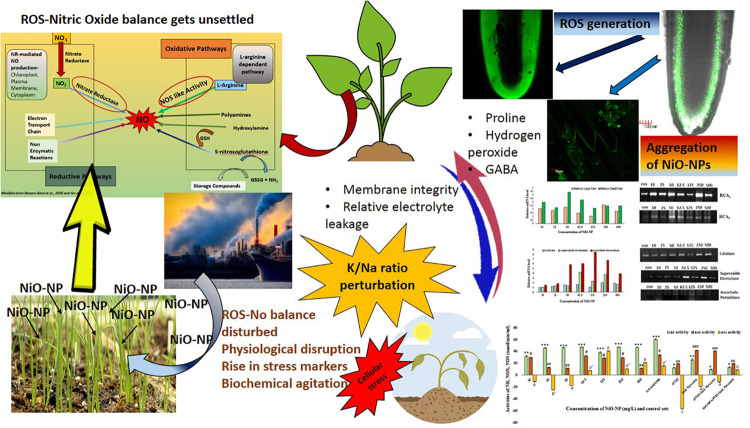
Diagrammatic view of the change in ROS–NO dynamics and the effect on cellular physiology.

In its entirety, it can be stated that NiO-NP exposure in *A. cepa* roots does indeed lead to oxidative stress, by way of perturbed production and dissociation of various reactive species, especially RNS (NO) and ROS (H_2_O_2_) (Figure 8). The unique properties of NiO-NP initiate a compelling duel between intracellular ROS, NO production, and the ameliorating antioxidant systems present in the plant tissues. This distinctive interaction is unique to this particular nanoparticle and the biotic component and is aided by the surrounding biophysical features like temperature, time of exposure, the dissociation constant, and aggregate formation. Different cellular cascades were individually discomposed and those in turn affected the entire abiotic stress response system of the exposed plant; the most affected are the antioxidant systems headed by CAT. Different mechanisms of NO production cascades were affected as well, which was documented by upregulated transcripts of antioxidants and higher activities of key enzymes. At higher concentrations, this ENP induced overwhelming oxidative stress and superfluous ROS accumulation, with a NO upsurge that was beyond restoration by the existing cellular mechanism, thus eventually leading to suspension of cellular activities and cell death. Further investigations on cellular signal transduction and signal cascades are necessary to understand the exact mechanism of this physiological collapse in cells subjected by NiO-NP. Such studies in a metallic nanoparticle-treated plant are still in preliminary stages and the present body of work could add up to the current bursar of existing knowledge in ROS–NO interaction in plants and help plant biologists to have a better understanding of ROS–NO dynamics in stressed tissue (Xiong et al., 2010). Additionally, further work to detect the gene-level cascades operating in case of NiO-NP-induced stress should be carried out, which will help in understanding the core mechanism of plants’ retaliation to heavy metals and engineered nanoparticles.

## Data Availability Statement

The raw data supporting the conclusions of this article will be made available by the authors, without undue reservation.

## Author Contributions

All authors listed have made a substantial, direct and intellectual contribution to the work, and approved it for publication.

## Conflict of Interest

The authors declare that the research was conducted in the absence of any commercial or financial relationships that could be construed as a potential conflict of interest.

## Footnotes

1 https://www.atsdr.cdc.gov/phs/phs.asp?id=243&tid=44

2 https://monographs.iarc.fr/wp-content/uploads/2018/06/mono100C-10.pdf

3 WHO,2000; http://www.euro.who.int/__data/assets/pdf_file/0014/123080/AQG2ndEd_6_10Nickel.pdf

4 https://www.oecd.org/chemicalsafety/testing/33653757.pdf

## Abbreviations

ENPs: engineered nanoparticles
NiO-NP: nickel oxide nanoparticles
ROS: reactive oxygen species
RNS: reactive nitrogen species
CAT: catalase
APX: ascorbate peroxidase
SOD: superoxide dismutase
RCA: Rubisco activase
NOS: nitric oxide synthase
NR: nitrate reductase
NOX: NADPH oxidase
NO: nitric oxide
H_2_O_2_: hydrogen peroxide
K/Na ratio: potassium:sodium ratio
*A. cepa*: *Allium cepa*
EMS: ethyl methanesulfonate
cPTIO: 2-(4-carboxyphenyl)-4,4,5,5-tetramethylimidazoline-1-oxyl-3-oxide potassium salt
Sod. Pyr.: sodium pyruvate
FITC: fluorescein isothiocyanate
DMF: dimethylformamide
APTS: aminopropyl triethoxysilane
CLSM: confocal laser scanning microscope
DAF-FM-DA: 4-amino-5-methylamino-2 ′,7 ′ -difluorofluorescein diacetate
PBS: phosphate-buffered saline
GABA: gamma amino butyric acid
DTT: dithiothreitol
TCA: trichloroacetic acid
KI: potassium iodide
ICP-OES: inductively coupled plasma-optical emission spectroscopy
UNEP: United Nations Environment Programme
USEPA: United States Environmental Protection Agency
OECD: Organization for Economic Co-operation and Development
WHO: World Health Organization
CuO-NP: copper oxide nanoparticle
RBOHs: respiratory burst oxidase homologs
RuBP: Rubisco
