# Supplementary material for: Effect of Engineered Nickel Oxide Nanoparticle on Reactive Oxygen Species–Nitric Oxide Interplay in the Roots of Allium cepa L
Source: Front Plant Sci. 2021 Feb 9;12:586509. doi: 10.3389/fpls.2021.586509 (PMC7901573; doi:10.3389/fpls.2021.586509)
Supplement: Supplementary file 1 [file Data_Sheet_1.DOCX]

| Set | NiO-NO  (mg L-1) | Ethyl methane sulfonate (EMS)/ 1% Triton X | cPTIO | Sodium pyruvate | DD water | H_2_O_2_ |
| --- | --- | --- | --- | --- | --- | --- |
| NC | -- | -- | -- | -- | Yes | -- |
| T1 | 10 | -- | -- | -- | -- | -- |
| T2 | 25 | -- | -- | -- | -- | -- |
| T3 | 50 | -- | -- | -- | -- | -- |
| T4 | 62.5 | -- | -- | -- | -- | -- |
| T5 | 125 | -- | -- | -- | -- | -- |
| T6 | 250 | -- | -- | -- | -- | -- |
| T7 | 500 | -- | -- | -- | -- | -- |
| C1 | -- | 0.4 mM | -- | -- | -- | -- |
| C2 | -- | -- | -- | 10 mM | -- | -- |
| C3 | -- | -- | 100µM | -- | -- | -- |
| C4 | -- | -- | -- | -- | -- | 25 µM |
| C5 | -- | -- | 100µM | 10 mM | -- | -- |
| C6 | 125 | -- | 100µM | 10 mM | -- | -- |
| C7 | 125 | -- | 100µM | 10 mM | -- | 25 µM |
| C8 | 125 | 0.4 mM | 100μM | 10mM | -- | 25μM |

Supplementary Table 1 showing the composition of various experimental sets


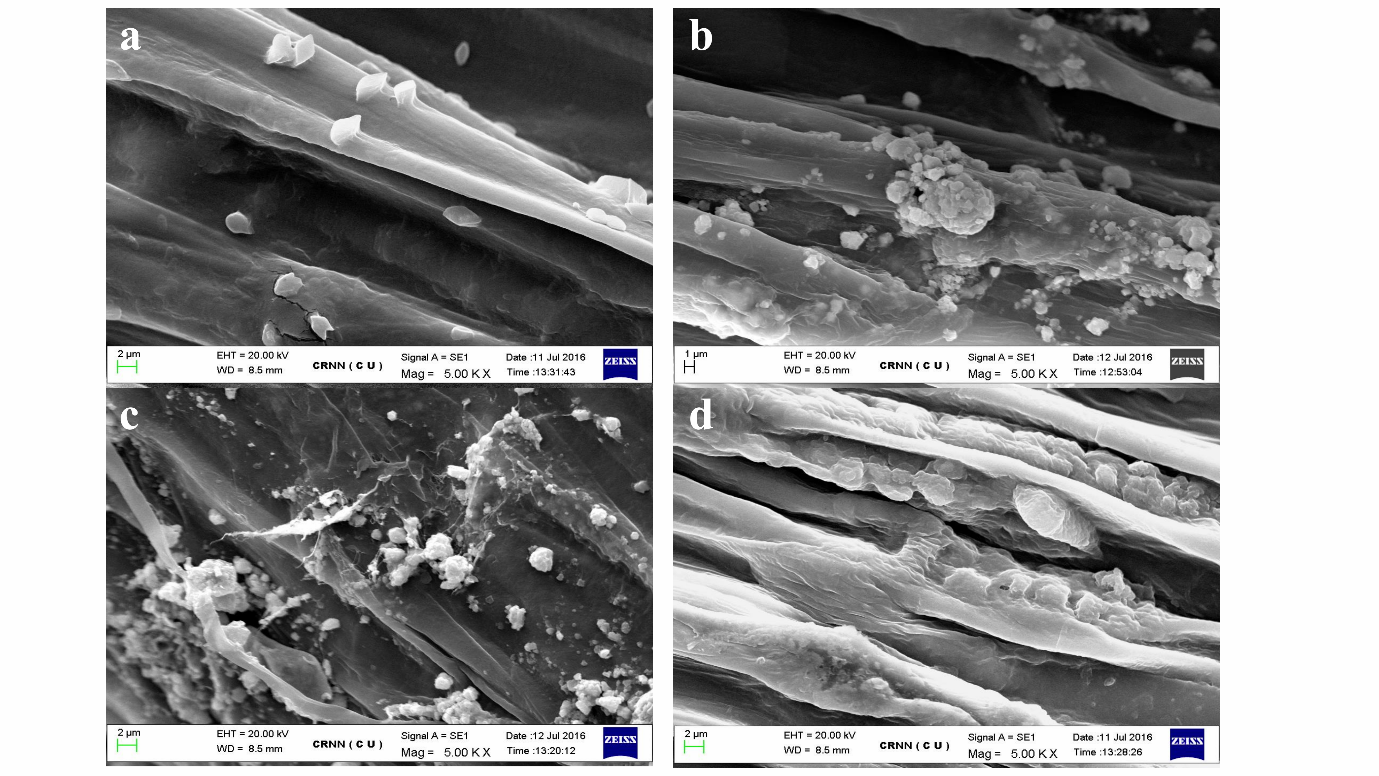


Supplementary Figure 1: SEM images showing clumping of nanoparticles in a dose dependent manner on root tips of *A. cepa* after 24 hours of treatment– (a)- 25 mg L^-1^ (b)- 62.5 mg L^-1^ (c)- 125 mg L^-1^ (d)- 500 mg L^-1^

The above diagrams show how NiO-NPs formed aggregation on to the outer cell surface, gradually forming larger clumps and causing extensive damages.


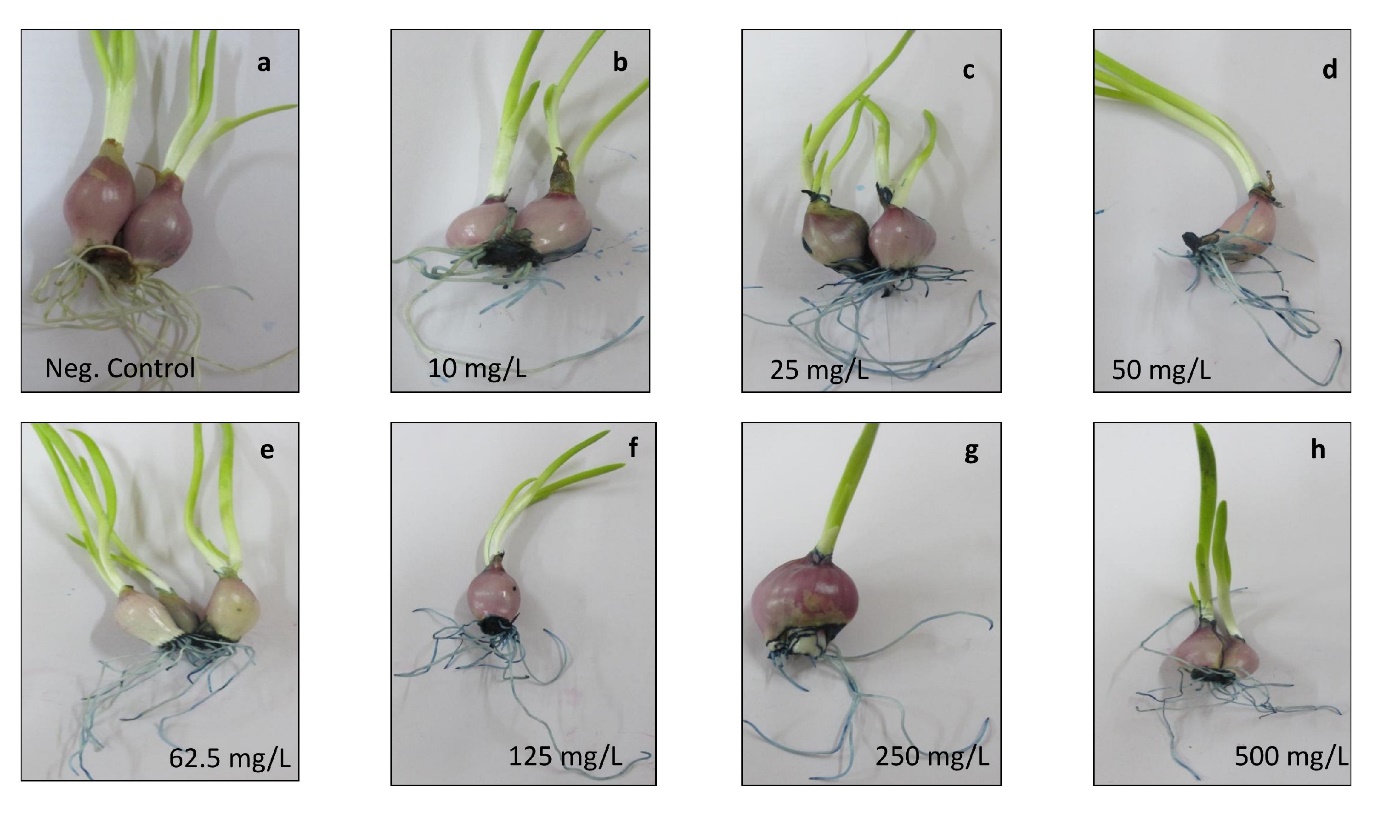


Supplementary Figure 2- Root tips stained with Evans blue showing extent of damage to cell membrane (a- negative control, b- tissue exposed to 10 mg L^-1^, c- tissue exposed to 25 mg L^-1^, d- tissue exposed to 50 mg L^-1^, e- tissue exposed to 62.5 mg L^-1^, f- tissue exposed to 125 mg L^-1^, g- tissue exposed to 250 mg L^-1^, h- tissue exposed to 500 mg L^-1^)


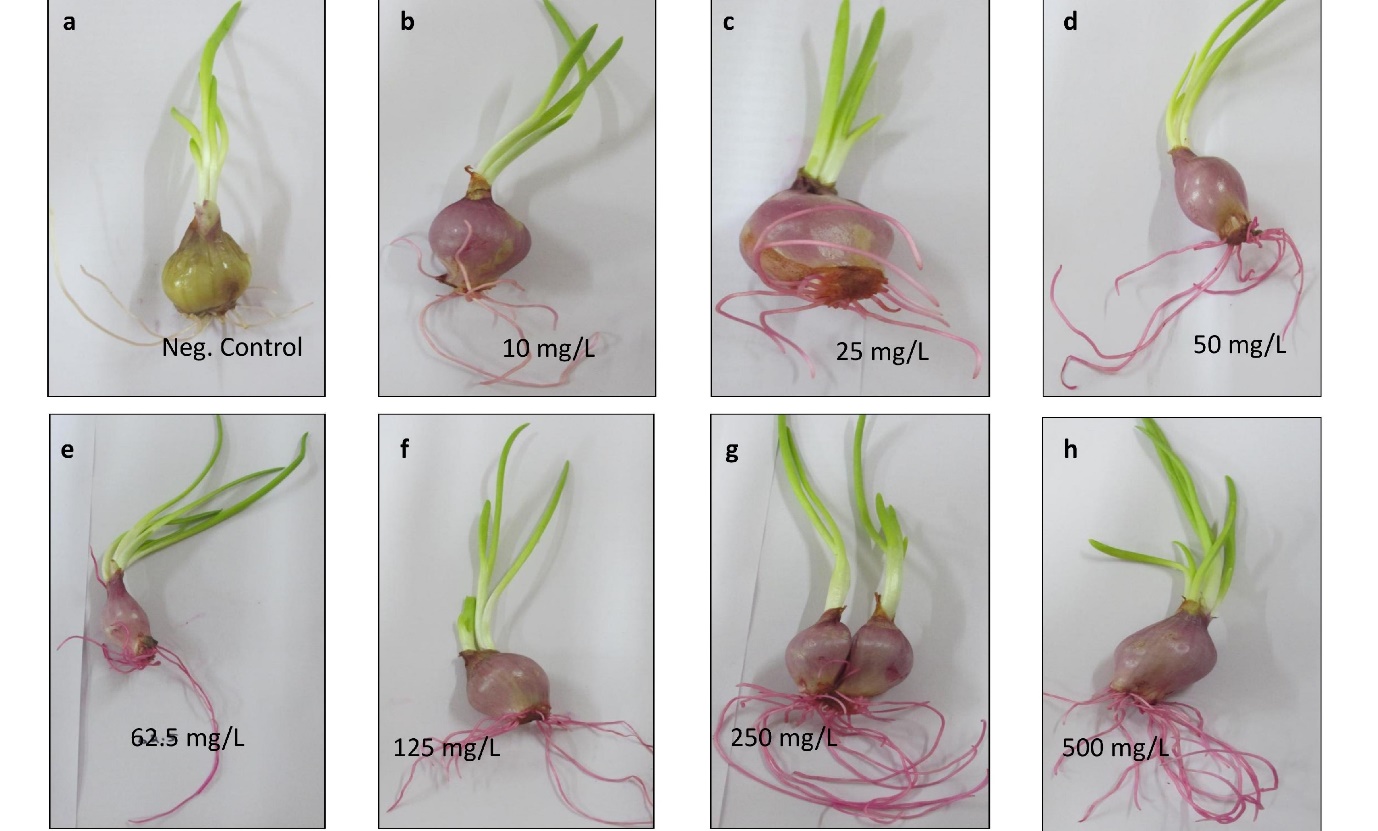


Supplementary Figure 3- Root tips stained with Schiff’s Reagent showing extent of lipid peroxidation (a- negative control, b- tissue exposed to 10 mg L^-1^, c- tissue exposed to 25 mg L^-1^, d- tissue exposed to 50 mg L^-1^, e- tissue exposed to 62.5 mg L^-1^, f- tissue exposed to 125 mg L^-1^, g- tissue exposed to 250 mg L^-1^, h- tissue exposed to 500 mg L^-1^

Supplementary Table 2- table showing the forward and reverse sequences of the primers used

| Primer | Forward sequence | Reverse sequence |
| --- | --- | --- |
| 1.RCA large | 5'-CGTGACGGGCGTATGGAGAAG-3' | 5'-GCACGAA  GAGCGCCGAAGAA ATC-3’ |
| 2.RCA small | 5'-TTCTGCGCCATCCAGCTG  AA-3' | 5'-CCTCCTCCTCCTATGCA GG-3' |
| 3. Catalase | 5′-AGCCGGTGGGAAGATTAGTT-3′ | 5′-AAGCAAGCTTTTGACCCAGA-3’ |
| 4. Superoxide Dismutase | 5’- AAGATGGAGATGCACCAACC-3’ | 5’- CCAGCATTTCCAGTGGTTTT-3’ |
| 5. Ascorbate Peroxidase | 5’-GTTCAAGGCTGAGCAAGGAC -3′ | 5’-GGGGATTGGTAGTCCAAGGT  -3′ |
| 6. Actin | AGAGCAGTATTCCCAAGC | TCTTCAGGAGCAACACGA |

Supplementary Figure 4-Relative mRNA level of reference gene used (Actin)
